# Genetic Deletion or Pharmacological Inhibition of Cyclooxygenase-2 Reduces Blood-Brain Barrier Damage in Experimental Ischemic Stroke

**DOI:** 10.3389/fneur.2020.00887

**Published:** 2020-08-20

**Authors:** Changjun Yang, Yi Yang, Kelly M. DeMars, Gary A. Rosenberg, Eduardo Candelario-Jalil

**Affiliations:** ^1^Department of Neuroscience, McKnight Brain Institute, University of Florida, Gainesville, FL, United States; ^2^Department of Neurology, Center for Memory and Aging, University of New Mexico Health Sciences Center, Albuquerque, NM, United States

**Keywords:** ischemic stroke, blood-brain barrier, cyclooxygenase-2, matrix metalloproteinase-9, tight junction proteins

## Abstract

Cyclooxygenase (COX)-2 and matrix metalloproteinase (MMP)-9 are two crucial mediators contributing to blood-brain barrier (BBB) damage during cerebral ischemia. However, it is not known whether MMP-9 activation is involved in COX-2-mediated BBB disruption in ischemic stroke. In this study, we hypothesized that genetic deletion or pharmacological inhibition of COX-2 reduces BBB damage by reducing MMP-9 activity in a mouse model of ischemic stroke. Male COX-2 knockout (COX-2^−/−^) and wild-type (WT) mice were subjected to 60 min of middle cerebral artery occlusion (MCAO) followed by 24 h of reperfusion. Genetic deletion of COX-2 or post-ischemic treatment with CAY10404, a highly selective COX-2 inhibitor, significantly reduced BBB damage and hemorrhagic transformation, as assessed by immunoglobulin G (IgG) extravasation and brain hemoglobin (Hb) levels, respectively. Immunoblotting analysis showed that tight junction proteins (TJPs) zonula occludens (ZO)-1 and occludin as well as junctional adhesion molecule-A (JAM-A) and the basal lamina protein collagen IV were dramatically reduced in the ischemic brain. Stroke-induced loss of these BBB structural proteins was significantly attenuated in COX-2^−/−^ mice. Similarly, stroke-induced loss of ZO-1 and occludin was significantly attenuated by CAY10404 treatment. Ischemia-induced increase in MMP-9 protein levels in the ipsilateral cerebral cortex was significantly reduced in COX-2^−/−^ mice. Stroke induced a dramatic increase in MMP-9 enzymatic activity in the ischemic cortex, which was markedly reduced by COX-2 gene deficiency or pharmacological inhibition with CAY10404. Levels of myeloperoxidase (MPO, an indicator of neutrophil infiltration into the brain parenchyma), neutrophil elastase (NE), and lipocalin-2 (LCN2, also known as neutrophil gelatinase-associated lipocalin), measured by western blot and specific ELISA kits, respectively, were markedly increased in the ischemic brain. Increased levels of markers for neutrophil infiltration were significantly reduced in COX-2^−/−^ mice compared with WT controls following stroke. Altogether, neurovascular protective effects of COX-2 blockade are associated with reduced BBB damage, MMP-9 expression/activity and neutrophil infiltration. Our study shows for the first time that MMP-9 is an important downstream effector contributing to COX-2-mediated neurovascular damage in ischemic stroke. Targeting the COX-2/MMP-9 pathway could represent a promising strategy to reduce neuroinflammatory events in order to preserve the BBB integrity and ameliorate ischemic stroke injury.

## Introduction

Prostaglandin-endoperoxide synthases (PTGS), also known as cyclooxygenases (COXs), are key players in inflammation, and are targets of the widely used non-steroidal anti-inflammatory drugs (NSAIDs) (1). COX exists in three forms: COX-1,−2, and−3, where COX-1 and COX-3 (a splice variant of COX-1) are encoded by the same *PTGS1* gene, and COX-2 is encoded by the *PTGS2* gene (1). COX-1 is constitutively expressed in most tissues and largely contributes to normal physiological functions. COX-2 is an inducible enzyme that is mostly associated with pathophysiological functions in response to proinflammatory stimuli or growth factors (1), whereas COX-3 is not functional in humans (2).

COX-2 inhibition is an attractive pharmacological target since the metabolism of arachidonic acid (AA) through the COX pathway produces large amounts of proinflammatory prostanoids, such as prostaglandin E_2_ (PGE_2_), which are key inflammatory mediators (3, 4). In ischemic rat brain, a dramatic increase in COX-2 expression has been reported starting at 30 min and lasting up to 15 days after stroke (5–8). Previous findings from our group and others have demonstrated that pharmacological inhibition of COX-2 confers neuroprotection in ischemic brain injury (9–14). COX-2 knockout (COX-2^−/−^) mice display a dramatic reduction in the susceptibility to excitotoxicity and ischemic brain injury (15, 16), whereas neuronal overexpression of COX-2 increases infarct volume associated with a dramatic increase in PGE_2_ levels in the ischemic brain (17). The detrimental effect of COX-2 after cerebral ischemia is attributed to the production of PGE_2_ rather than to the generation of oxidative stress (18). Similarly, we previously found that the increase in PGE_2_ formation in the ischemic cortex correlates with the evolution of cerebral infarct in this brain region, and the accumulation of PGE_2_ in the ischemic cerebral cortex paralleled the substantial increase in blood-brain barrier (BBB) breakdown and leukocyte infiltration (12). These findings are in line with a previous report showing that PGE_2_ produces a marked BBB breakdown when administered intracerebrally in rats (19).

There is increasing evidence indicating that COX-2 derived PGE_2_, matrix metalloproteinase (MMP)-9 production and BBB disruption are important components of neuronal death in the ischemic penumbra, a potentially salvageable tissue surrounding the infarct core (6, 17, 20, 21). Active MMP-9 degrades the basal lamina around blood vessels and vascular endothelial tight junction proteins (TJPs), leading to disruption of the BBB (22–24). This opening of the BBB is associated with vasogenic edema and infiltration of polymorphonuclear leukocytes, which exacerbate brain injury. In non-neural cell types, such as tumor cells, COX-2-derived PGE_2_ increases MMP-9 expression and activity. It has been demonstrated that tumor cells invasion or metastasis are dramatically reduced by COX-2 inhibitors, and this effect is mediated by a significant reduction in PGE_2_-mediated MMP-9 expression/activity (25–28). However, it is not known whether MMP-9 activation is involved in COX-2-mediated BBB disruption in ischemic stroke.

In this study, we hypothesized that genetic deletion or pharmacological inhibition of COX-2 reduces BBB damage by reducing MMP-9 activity in a mouse model of ischemic stroke. We found that gene deficiency of COX-2 or post-ischemic treatment with the COX-2 inhibitor, CAY10404, significantly reduced BBB damage and hemorrhagic transformation following transient focal cerebral ischemia, and these neurovascular protective effects were associated with a significant decrease in MMP-9 activity, reduced neutrophil infiltration, and a preservation of structural proteins composing the neurovascular unit.

## Methods

### Animals

All animal procedures were approved by the University of Florida Institutional Animal Care and Use Committee (protocol # 201607934) and performed in accordance with the NIH Guide for the Care and Use of Laboratory Animals, the ARRIVE guidelines (https://www.nc3rs.org.uk/arrive-guidelines). Mice were housed five per cage with free access to food and water in a temperature- and humidity-controlled environment with a 12-h light/dark cycle. All mice were acclimated to our animal facility for at least 7 days before any surgical procedure and efforts were made to minimize animal suffering and the number of animals used.

### Induction of Transient Focal Cerebral Ischemia and Drug Treatment

Male PTGS2 knockout (referred in this manuscript as COX-2^−/−^) and wild-type mice on a C57BL/6 background were obtained from Taconic. Focal cerebral ischemia was induced by occlusion of right middle cerebral artery (MCA) using a silicone-coated filament as described previously by our group (29, 30). Briefly, mice (10–12 weeks old) were anesthetized with 3% isoflurane and surgical levels of anesthesia were maintained by inhalation of 1.5–2% isoflurane in medical grade oxygen. The right common carotid artery (CCA), external carotid artery (ECA), and internal carotid artery (ICA) were exposed via a midline vertical incision in the anterior neck. The CCA was ligated with a 6-0 silk suture at the proximal portion from the carotid bifurcation. A 12 mm length of a 6-0 silicone-coated nylon filament (Doccol, Cat. No. 602123) was inserted into the CCA and advanced gently into the ICA ~9–10 mm from the carotid bifurcation until a mild resistance was felt and cerebral blood flow (CBF) was reduced by at least 75% of the baseline value, as assessed by laser Doppler flowmetry. After 60 min of MCA occlusion, the filament was gently retracted to allow reperfusion. The skin was closed and anesthesia was discontinued, and the mice were allowed to recover in a temperature-controlled chamber. Sham-operated animals received the same surgical procedures except for the MCA occlusion. For the treatment with vehicle or CAY10404 (Cat. No. 70210, Cayman Chemical, Ann Arbor, MI), a highly selective COX-2 inhibitor, wild-type mice were injected with a single dose of vehicle (0.1% DMSO in 10% Solutol dissolved in physiological saline) or CAY10404 (10 mg/kg; i.p.) at the start of reperfusion. The selectivity of CAY10404 toward COX-2 is comparable to the selectivity index of second-generation selective COX-2 inhibitors, such as valdecoxib and etoricoxib (31). This dose of CAY10404 was selected based on previous reports showing antinociceptive and anti-inflammatory effects in mice (32, 33).

### Measurement of Infarct Volume

Brain infarct volume was measured using 2,3,5-triphenyltetrazolium chloride (TTC) staining as described in detail in our previous studies (29, 34). Mice were euthanized at 24 h post-MCAO and brains were collected after transcardiac perfusion with ice-cold saline. The brain was placed in a slicing matrix (Zivic Instruments, Pittsburgh, PA, USA) and sliced into six 1-mm thick coronal sections. Sections were stained with 2% TTC in phosphate-buffered saline (PBS) for 30 min at room temperature followed by fixation with 4% paraformaldehyde (PFA) in PBS, then scanned at 600 dpi by a HP Scanjet 8300 scanner (Palo Alto, CA). Infarct volume corrected for edema was determined with Adobe Photoshop as we have previously reported (29, 34–37).

### Protein Extraction From Brain Tissue

At 24 h after MCAO, mouse brains were dissected out after transcardiac perfusion with ice-cold saline, and ipsilateral and contralateral cerebral cortices were collected on dry ice and immediately stored at −80°C until further processing. Cortical tissue was homogenized in radioimmunoprecipitation (RIPA) lysis buffer containing protease and phosphatase inhibitors as detailed in our previous report (29). Protein concentration in resulting tissue homogenates was determined by Pierce™ BCA assay kit (Cat. No. 23227, Thermo Scientific, Rockford, IL) and samples were aliquoted and stored at −80°C until use.

### Immunoblotting Analysis

Fifty micrograms of protein were separated on 4–20% SDS-polyacrylamide gels and then transferred onto nitrocellulose membranes. Membranes were then blocked for 1 h at room temperature with 5% non-fat milk in Tris-buffered saline. Thereafter, the membranes were incubated at 4°C overnight with primary mouse anti-IgG antibody (1:2,000; Cat. No. 7076; Cell Signaling Technology), rabbit anti-ZO-1 antibody (1:500; Cat. No. 61-7300; Invitrogen), rabbit anti-occludin antibody (1:500; Cat. No. 61-7300; Invitrogen), rabbit anti-JAM-A antibody (1:5,000; Cat. No. ab52647; Abcam), goat anti-Collagen IV antibody (1:500; Cat. No. 1340-01; Southern Biotech), rabbit anti-MMP-9 antibody (1:1,000; Cat. No. sc-6841R; Santa Cruz Biotechnology), rabbit anti-MPO antibody (1:1,000; Cat. No. sc-16128-R; Santa Cruz Biotechnology), or mouse anti-β-actin antibody (1:10,000; Cat. No. A1978; Sigma). The membranes were then washed with TBST three times at 5 min intervals, incubated with goat anti-rabbit IRDye 800CW (1:30,000; Li-Cor, Lincoln, NE), donkey anti-mouse IRDye 680LT (1:40,000; Li-Cor), or donkey anti-goat IRDye 800CW (1:30,000; Li-Cor) secondary antibodies for 1 h at room temperature. Immunoreactive bands were visualized and densitometrically analyzed using Odyssey infrared scanner and Image Studio 2.0 software (Li-Cor).

### ELISA

Levels of IgG and hemoglobin (Hb) in the brain parenchyma were measured using commercial ELISA kits (Cat. Nos. E-90G and E-90HM, respectively; Immunology Consultants Laboratory, Inc.; Portland, OR) according to the manufacturer's instructions since extravasation of these plasma proteins into the brain tissue is a sensitive marker of BBB disruption after ischemia. A total of 50 μg protein extracted from ipsilateral and contralateral cerebral cortex of mouse brain were used for the IgG measurement, while 15 μg of total protein were used for the Hb measurement. All samples were assayed in duplicated and optical absorbance at 450 nm was measured with a Synergy™ HT Multi-Mode Plate Reader (Biotek Instruments, Winooski, VT).

Levels of neutrophil elastase (NE) and lipocalin-2 (LCN2, also known as neutrophil gelatinase-associated lipocalin) in ipsilateral and contralateral cerebral cortices were measured using specific ELISA kits purchased from TSZ Scientific (NE: Cat. No. M7718; Framingham, MA) and R&D Systems (LCN2: Cat. No. MLCN20; Minneapolis, MN), respectively. According to the manufacturer's instructions, a total of 50 μg of protein from ipsilateral and contralateral cortices of mouse brains were used for the NE measurement, while 40 μg of protein were used for the LCN2 measurement.

### Measurement of MMP-9 Enzymatic Activity

MMP-9 enzymatic activity in cortical homogenate was measured using an immunocapture assay method as reported in detail in our previous studies (29, 38). Briefly, 96-well high binding plates (Cat. No. 655077; Greiner Bio-One, Monroe, NC) were coated with MMP-9 antibody (Cat. No. sc-6841R; Santa Cruz Biotechnology, Dallas, TX) following protein A/G pre-coating. Then, a total of 50 μg protein extracted from mouse brain cerebral cortex was added to each well and incubated at 4°C overnight. After incubation, wells were washed with TCNB buffer (50 mM Tris, 10 mM CaCl_2_, 150 mM NaCl, 0.05% Brij-35) and 1 μM of 520 MMP FRET substrate III (Cat. No. 60570-01; AnaSpec, San Jose, CA) was added. Plates were incubated for 48 h at 37°C, then relative fluorescence units (RFUs) were read at excitation/emission wavelengths of 485/528 nm in a Synergy™ HT Multi-Mode Plate Fluorescence Reader. The average value from one paired substrate control wells was used to subtract baseline fluorescence from sample wells.

### Power Analysis, Randomization, Blinding, and Statistics

We performed an *a priori* sample size calculation using the G^*^Power v.3.1 software. Using means and standard deviations from our preliminary studies in this mouse ischemic stroke model, we calculated Cohen effect size (*d*). We compared two independent groups in a two-tailed unpaired *t*-test using α = 0.05, and β (type II error) of 0.2 with a power of 80%. The present study was powered with the expectation that we will detect a difference in plasma protein extravasation between genotypes of at least 25%. For the difference to become statistically significant, we calculated a sample size of *n* = 9 with an effect size of *d* = 1.4138. All mice were randomly allocated to experimental groups using the randomization tool developed by GraphPad Prism (http://www.graphpad.com/quickcalcs/randomize1.cfm). The investigators performing stroke surgeries, euthanizing animals, or performing outcome assessments (molecular biology analyses, infarct size calculation) had no knowledge of the genotype or treatment group to which an animal belonged. All values were expressed as mean ± standard deviation (SD). An independent unpaired Student's *t*-test was performed for comparison between two groups and a two-way ANOVA followed by Bonferroni post-tests for comparisons of multiple groups. GraphPad Prism 6 was used to conduct data analysis, and *P* < 0.05 was considered statistically significant.

## Results

### COX-2 Deficiency Significantly Reduces Stroke-Induced BBB Breakdown and Hemorrhagic Transformation

**Figure 1:**
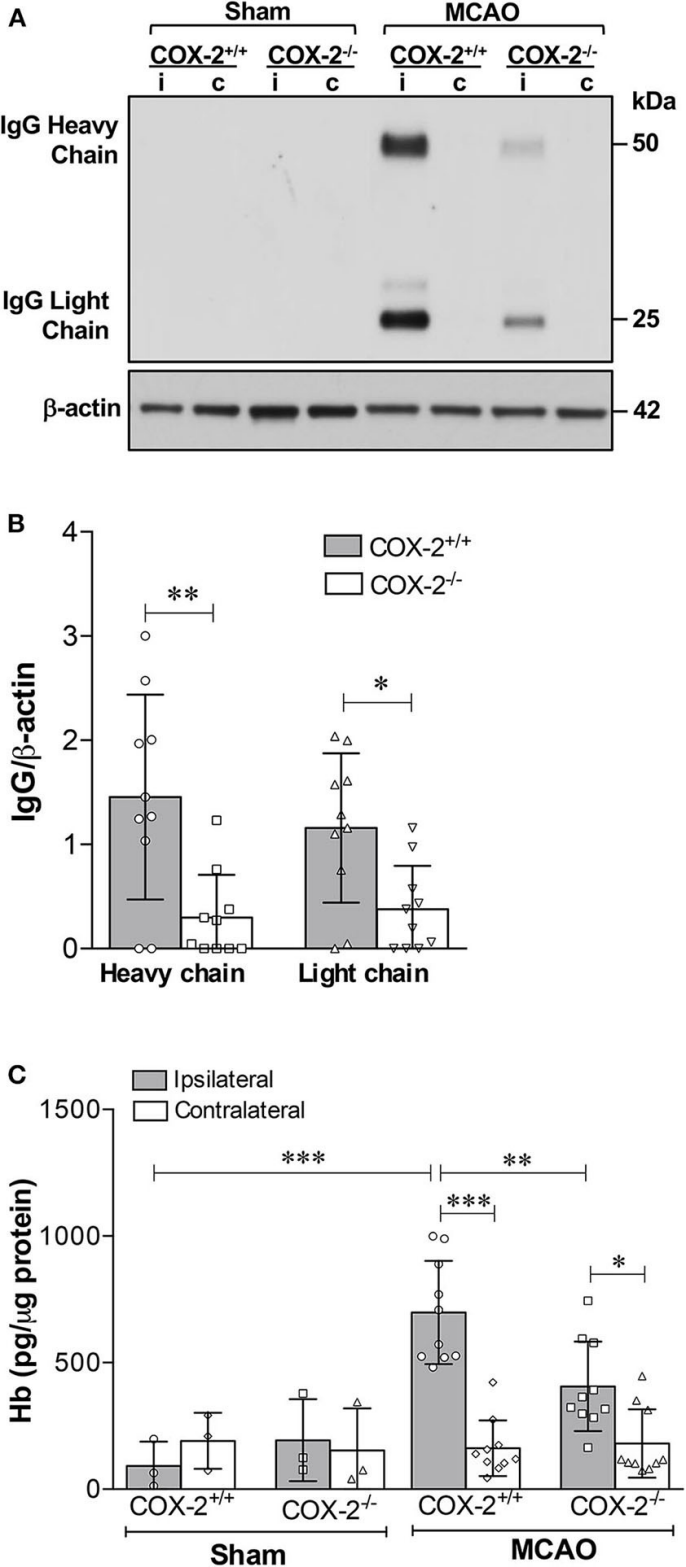
COX-2 genetic inactivation protects against stroke-induced BBB breakdown and hemorrhagic transformation. BBB permeability was assessed by immunoglobulin G (IgG) extravasation into the brain, as quantified by immunoblotting. **(A)** Representative western blot for IgG (heavy and light chains) in the cerebral cortex of wild-type (COX-2^+/+^) and knockout (COX-2^−/−^) mice; i, ipsilateral; c, contralateral. There is a significant reduction in BBB damage in COX-2^−/−^ mice compared to wild-type controls at 24 h following 60 min of temporary middle cerebral artery occlusion (MCAO). **(B)** Densitometric analysis of heavy and light chains of IgG normalized to β-actin in the ischemic cortex of mice of both genotypes. Unpaired *t*-test, *P < 0.05 and **P < 0.01. **(C)** Hemoglobin (Hb) levels in the brains were quantified using a highly sensitive ELISA as a measure of hemorrhagic transformation following ischemia. COX-2^−/−^ mice had a significant reduction in Hb levels after stroke compared to COX-2^+/+^ controls. Two-way ANOVA with Bonferroni post-tests. *P < 0.05, **P < 0.01, and ***P < 0.001. Bars represent mean ± SD. *n* = 10 in MCAO groups of each genotype. Sham-operated groups (*n* = 3).

Inflammatory responses significantly contribute to BBB breakdown, which is the main cause of hemorrhagic transformation after ischemic stroke (39). Since COX-2 actively participates in neuroinflammation after stroke (6, 7, 12), we sought to determine whether COX-2 deficiency lessens BBB breakdown and hemorrhagic transformation utilizing COX-2 null mice and their wild-type controls subjected to 60 min of MCAO followed by 24 h of reperfusion. To quantify BBB disruption, we measured levels of IgG extravasated into the brain using immunoblotting. In COX-2^+/+^ mice, IgG was dramatically increased in the ipsilateral cerebral cortex. BBB injury was largely reduced in COX-2^−/−^ mice compared to their wild-type controls, as demonstrated by significantly lower levels of both IgG chains in the ischemic brain (Figures 1A,B).

To examine whether COX-2 deficiency influences stroke-induced hemorrhagic conversion, we quantified hemoglobin (Hb) levels in brain homogenates utilizing a highly sensitive ELISA in both COX-2^+/+^ and COX-2^−/−^ mice at 24 h after ischemia. There was a significant reduction in Hb content in the ipsilateral cortex of COX-2^−/−^ mice compared with wild-type animals (Figure 1C). Collectively, these data suggest that COX-2 is an important mediator of BBB damage and hemorrhagic transformation following transient ischemic stroke.

### COX-2 Deficiency Ameliorates Stroke-Induced Damage to Structural Components of the Neurovascular Unit

**Figure 2:**
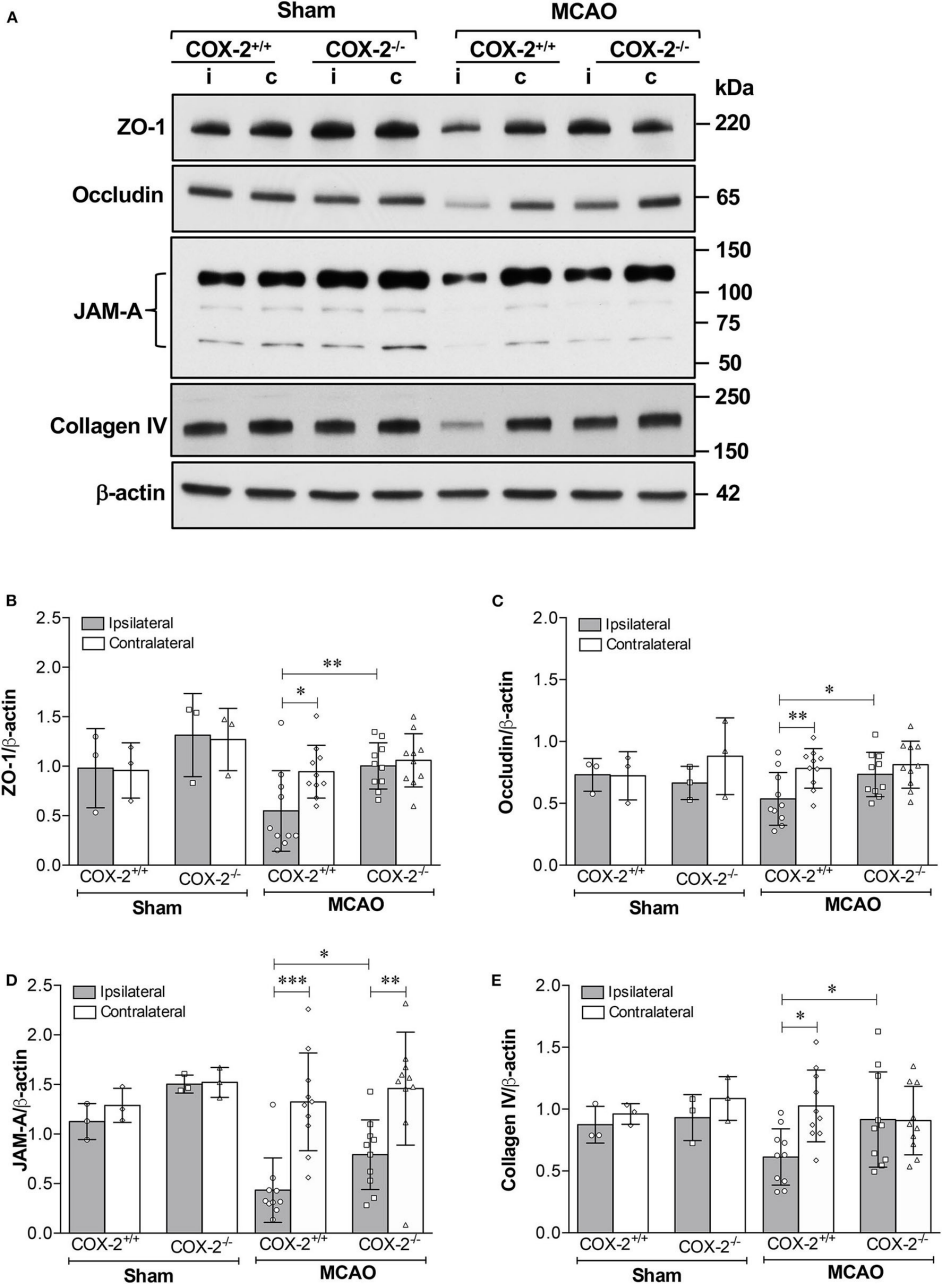
Reduced damage to BBB structural components in COX-2 deficient mice after stroke. Ischemia-induced loss of proteins composing the tight junctions, adherens junctions, and basal lamina significantly increases BBB permeability. COX-2 null mice displayed a reduced loss of ZO-1, occludin, JAM-A, and collagen IV after ischemic stroke compared to wild-type controls. **(A)** Representative immunoblot images for several BBB structural proteins. i, ipsilateral; c, contralateral. Densitometric analyses normalized to β-actin are presented for ZO-1 **(B)**, occludin **(C)**, JAM-A **(D)**, and collagen IV **(E)**. Two-way ANOVA with Bonferroni post-tests. *P < 0.05, **P < 0.01, and ***P < 0.001. Data are presented as mean ± SD. *n* = 10 in MCAO groups of each genotype. Sham-operated groups (*n* = 3).

The tight junction proteins (TJPs), ZO-1 and occludin, as well as the basal lamina protein, collagen IV, are critical for maintaining the integrity of the neurovascular unit (40, 41). Junctional adhesion molecule-A (JAM-A) is another BBB structural component that has been shown to facilitate tight junction assembly and modulate BBB permeability (42–44). Immunoblotting analysis of ZO-1, occludin, JAM-A, and collagen IV was performed in cortical homogenates of COX-2^+/+^ and COX-2^−/−^ mice subjected to 60 min of MCAO or sham surgery and 24 h of reperfusion. In the ipsilateral cerebral cortex of COX-2^+/+^ mice, levels of ZO-1, occludin, JAM-A, and collagen IV were markedly decreased after stroke. In COX-2^−/−^ mice, the damage to these BBB structural proteins was profoundly attenuated (Figure 2). This suggests that COX-2 deficiency protects against stroke-induced loss of proteins that compose the tight junctions and basal lamina. These findings help to explain the significant neurovascular protection seen in COX-2^−/−^ after ischemia.

### Genetic Deletion of COX-2 Reduces MMP-9 Protein Levels and Enzymatic Activity

**Figure 3:**
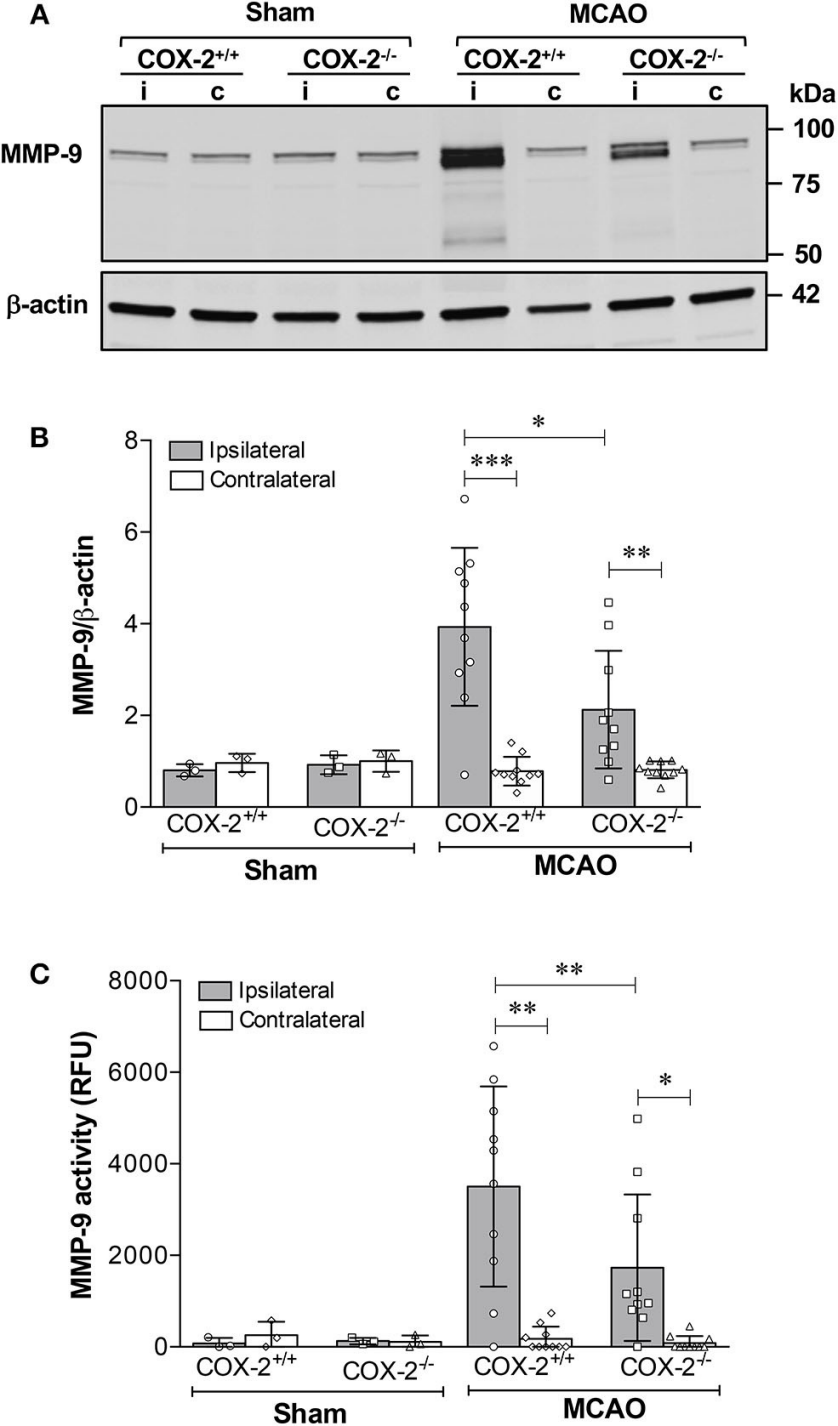
Genetic deletion of COX-2 significantly reduces MMP-9 activity and protein content in the ischemic brain. Transient focal cerebral ischemia followed by 24 h of reperfusion produced a dramatic increase in MMP-9 protein levels in the ipsilateral cerebral cortex as shown by immunoblotting. COX-2^−/−^ mice had a significant reduction in MMP-9 levels after ischemia compared with COX-2^+/+^. **(A)** Representative western blot for MMP-9 in cortical homogenates from ipsilateral (i) and contralateral (c) cortex. **(B)** Densitometric analysis of MMP-9 relative to β-actin. Reduced MMP-9 protein content was seen in COX-2^−/−^ compared with COX-2^+/+^ at 24 h following MCAO. **(C)** Fluorometric immunocapture assay of MMP-9 activity measured at 24 h in the cerebral cortex following transient ischemia showed a significant increase in COX-2^+/+^ mice (~8-fold increase with respect to contralateral). Compared with the wild-type, COX-2^−/−^ mice had a significant reduction in MMP-9 activity. For **(B,C)**, statistical analyses were performed using two-way ANOVA with Bonferroni post-tests. Significance was defined as *P < 0.05, **P < 0.01, and ***P < 0.001. Data are presented as mean ± SD. *n* = 10 in ischemic groups of each genotype. Sham-operated groups (*n* = 3).

MMP-9 is a potent proteinase involved in the degradation of extracellular matrix and TJPs, thus resulting in the BBB disruption (21, 23). MMP-9 protein levels and its activity in cerebral cortex were measured using immunoblotting and fluorometric immunocapture assay methods. As shown in Figures 3A,B, MMP-9 levels in the ipsilateral cortex of COX-2^+/+^ and COX-2^−/−^ mice were both dramatically increased after stroke, but much lower levels of MMP-9 were observed in COX-2^−/−^ mice compared to the wild-type animals. Similarly, stroke resulted in a dramatic increase in MMP-9 activity in the ipsilateral cortex of COX-2^+/+^ mice, and this increase was significantly attenuated in COX-2^−/−^ mice (Figure 3C). These findings suggest that COX-2 is involved in MMP-9-mediated BBB damage in ischemic stroke.

### COX-2 Deficient Mice Show Less Neutrophil Infiltration and Reduced Protein Levels of Neutrophil Elastase (NE) and Lipocalin-2 (LCN2) After Experimental Stroke

**Figure 4:**
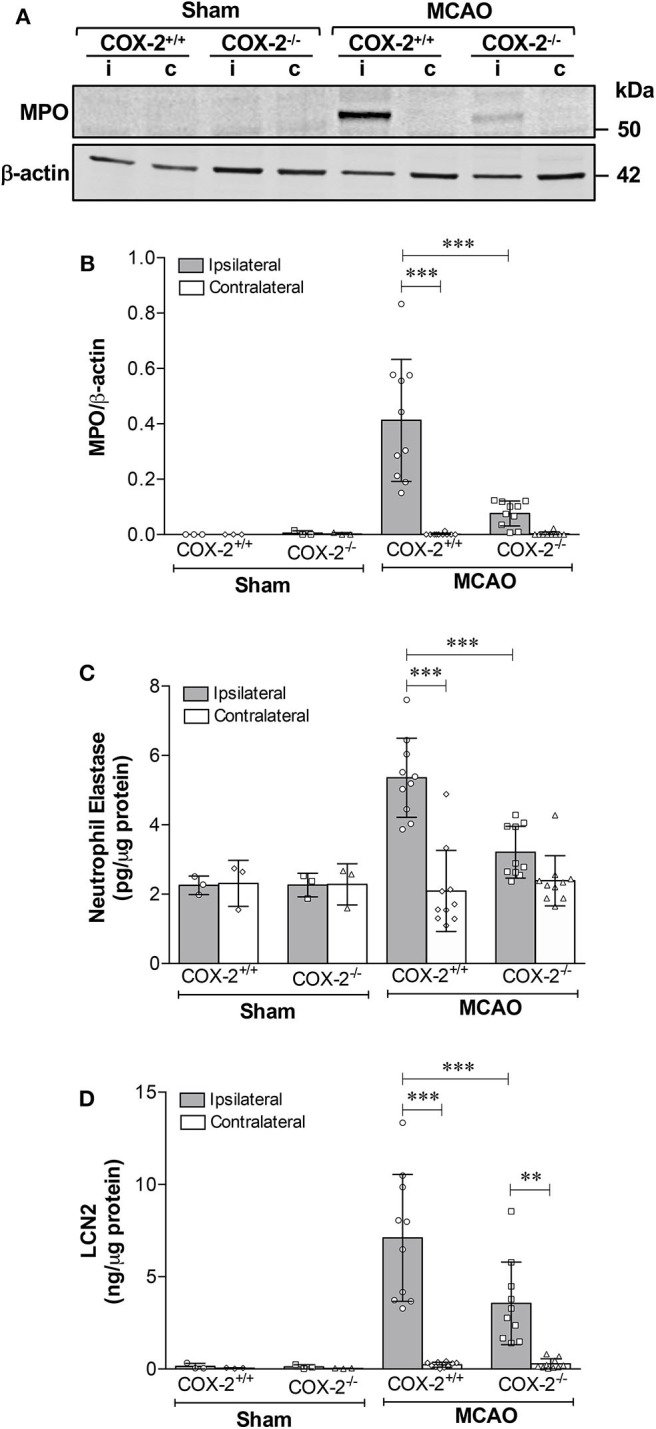
COX-2 deficiency results in less neutrophil infiltration and reduced protein levels of neutrophil elastase and lipocalin-2. Transient MCAO followed by 24 h of recirculation produced a significant infiltration of neutrophils as measured by myeloperoxidase (MPO) protein levels in the ischemic cerebral cortex. Genetic deletion of COX-2 prevented stroke-induced increase in MPO, which is suggestive of less neutrophil infiltration into the injured brain. **(A)** Representative western blot for MPO in homogenates from ipsilateral (i) and contralateral (c) cerebral cortices. **(B)** Densitometric analysis of MPO normalized to β-actin. **(C)** Neutrophil elastase protein levels measured by ELISA in cortical homogenates from COX-2^+/+^ and COX-2^−/−^ following ischemic stroke. Ischemia resulted in a significant increase in neutrophil elastase, a protease actively involved in BBB damage, in the ipsilateral cortex after 24 h of reperfusion. COX-2 deficient mice had a significant reduction in neutrophil elastase levels after ischemia compared to wild-type controls. **(D)** Lipocalin-2 (LCN2), also known as neutrophil gelatinase-associated lipocalin (NGAL), was dramatically increased in the ischemic brain following ischemia, as quantified using an ELISA kit. Compared to wild-type, COX-2^−/−^ mice had a significant reduction in LCN2 protein levels after stroke. Statistical analyses were performed using two-way ANOVA with Bonferroni post-tests. Significance was defined as **P < 0.01 and ***P < 0.001. Data are presented as mean ± SD. *n* = 10 in MCAO groups of each genotype. Sham-operated groups (*n* = 3).

To examine whether COX-2 deficiency affects neutrophil infiltration in ischemic stroke, levels of myeloperoxidase (MPO, an indicator of neutrophil infiltration into the brain parenchyma), neutrophil elastase (NE), and lipocalin-2 (LCN2, also known as neutrophil gelatinase-associated lipocalin) were measured in cortical homogenates of COX-2^+/+^ and COX-2^−/−^ mice at 24 h after MCAO. Immunoblotting and densitometric analysis show that MPO protein levels were dramatically induced by stroke in the ipsilateral cortex of COX-2^+/+^ mice, and very low levels of MPO were detected in the COX-2^−/−^ mice compared to the wild-type animals (Figures 4A,B). In line with these observations, levels of NE and LCN2 as measured by specific ELISA kits, in the ipsilateral cortex of COX-2^−/−^ mice were dramatically reduced after stroke compared to the wild-type animals (Figures 4C,D). Together, these data suggest that COX-2 contributes to neutrophil infiltration into the injured brain during ischemic stroke.

### Selective Pharmacological Inhibition of COX-2 Reduces Ischemia-Induced Neurovascular Injury and MMP-9 Activity

**Figure 5:**
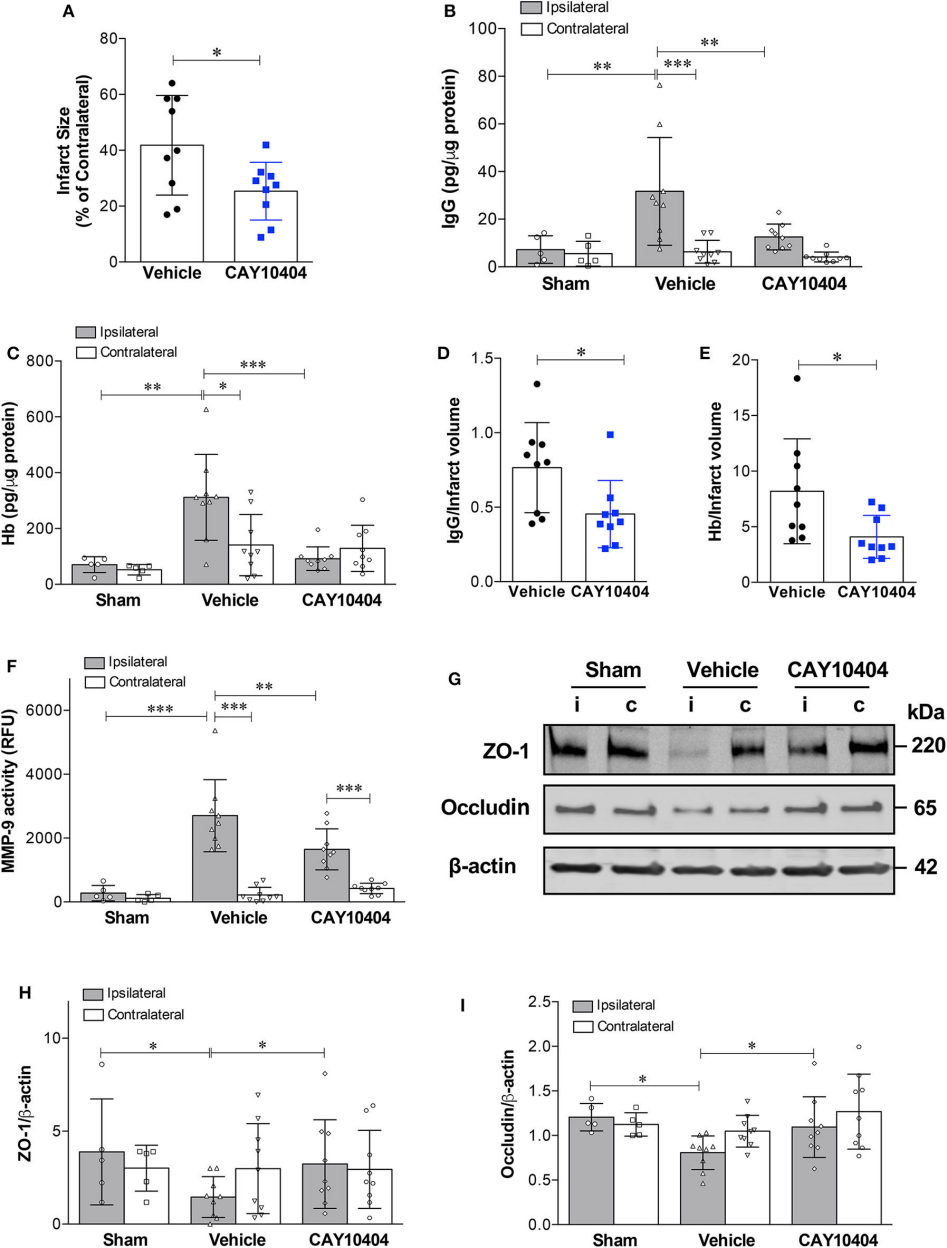
Pharmacological inhibition of COX-2 protects against stroke-induced BBB damage, hemorrhagic transformation, increased MMP-9 activity, and loss of tight junction proteins. CAY10404, a highly selective COX-2 inhibitor, was administered intraperitoneally at the start of reperfusion to wild-type mice subjected to MCAO. Ischemic controls received the vehicle. **(A)** Treatment with CAY10404 significantly reduced infarct size at 24 h after stroke. Unpaired *t*-test, *P < 0.05. **(B)** Extravasation of IgG into the ischemic cerebral cortex was significantly reduced by post-ischemic CAY10404 administration. Two-way ANOVA with Bonferroni *post-hoc* test. **P < 0.01 and ***P < 0.001. **(C)** Reduced hemorrhagic transformation in CAY10404-treated mice at 24 h after stroke, as quantified by brain hemoglobin (Hb) levels. Two-way ANOVA followed by Bonferroni *post-hoc* test. *P < 0.05, **P < 0.01, and ***P < 0.001. **(D)** COX-2 selective inhibition with CAY10404 reduced relative IgG extravasation normalized to the infarct size. Unpaired *t*-test, *P < 0.05. **(E)** Reduced Hb levels in CAY10404 group remained statistically significant after correcting for infarct size. Unpaired *t*-test, *P < 0.05. **(F)** A stark increase in MMP-9 activity was found in the ipsilateral cerebral cortex at 24 h following 60 min of experimental ischemic stroke. Treatment with the COX-2 inhibitor, CAY10404, significantly reduced MMP-9 activity compared with mice receiving the vehicle. Two-way ANOVA with Bonferroni *post-hoc* test. **P < 0.01 and ***P < 0.001. **(G)** Representative immunoblot images for the tight junction proteins, ZO-1 and occludin, in ipsilateral (i) and contralateral (c) cortices at 24 h post-MCAO. Densitometric analyzes of ZO-1 **(H)** and occludin **(I)** levels showed that CAY10404 treatment significantly protected against stroke-induced loss of these two tight junction proteins. Two-way ANOVA with Bonferroni *post-hoc* test, *P < 0.05. Data are presented as mean ± SD. Sham group (*n* = 5); Vehicle-treated (*n* = 9); CAY10404 group (*n* = 9).

Data from our experiments utilizing COX-2^−/−^ mice suggest that increased COX-2 activity is a key pathological event in stroke-induced neurovascular injury. However, there might be compensatory changes in germline COX-2^−/−^ mice, which could explain decreased BBB damage after stroke. To address this potential limitation of our genetic knockout experiments, we utilized a different experimental approach using the pharmacological COX-2 inhibitor, CAY10404. Similar to COX-2 genetic deletion, post-ischemic treatment with the highly selective COX-2 inhibitor, CAY10404, significantly reduced stroke size (Figure 5A), IgG extravasation (Figure 5B) and hemorrhagic transformation (Figure 5C) compared to the vehicle-treated group. Since the treatment with the COX-2 inhibitor might lessen the extent of BBB damage by reducing infarct size, we corrected for this potential confounder by expressing the extent of BBB disruption relative to the infarct volume (Figures 5D,E). Thus, the BBB permeability is normalized to the infarct to correct for smaller lesion sizes in animals receiving CAY10404. After correcting for infarct volume, treatment with CAY10404 significantly reduced IgG and Hb levels in ischemic brain (Figures 5D,E), which further supports the vasculoprotective effects of COX-2 inhibition. In line with the BBB permeability data, stroke-induced loss of tight junction proteins including ZO-1 and occludin was significantly attenuated by CAY10404 treatment in the ischemic brain (Figures 5G–I), which were associated with significant reduction of MMP-9 activity (Figure 5F). Taken together, these findings suggest that genetic deficiency or pharmacological inhibition of COX-2 significantly reduces stroke-induced neurovascular injury possibly by reducing MMP-9-mediated proteolysis of BBB structural components.

## Discussion

In the present study, we examined the effects of COX-2 blockade in a mouse model of transient focal cerebral ischemia. We found that COX-2 deficient mice had a significant reduction of BBB damage, hemorrhagic transformation, and neutrophil infiltration as compared with wild-type mice after 60 min of MCAO and 24 h of reperfusion, where this neurovascular protection is associated with reduced MMP-9 expression/activity. To further rule out the potentially compensatory effects of COX-2 gene deficiency in mice following stroke, we also investigated the effects of CAY10404, a highly selective COX-2 inhibitor, on BBB damage, hemorrhagic transformation, and MMP-9 activity in wild-type mice subjected to focal ischemia. As a result, pharmacological inhibition of COX-2 with CAY10404 treatment resulted in reduced infarct volume, BBB damage, hemorrhagic transformation and MMP-9 activity. To our knowledge, this study provides the first experimental evidence implicating COX-2 as a key mediator in the cascade of events leading to increased MMP-9 expression and activation in the ischemic brain resulting in BBB damage.

COX-2 inhibition has emerged as a potential therapeutic strategy for cerebral ischemia, targeting critical pathophysiological events that exacerbate the initial brain damage triggered by the ischemic episode. Previous findings from our group and others have demonstrated that COX-2 selective inhibitors, such as nimesulide (9, 11, 12), rofecoxib (13), SC58236 (14, 17), and NS398 (18) exert beneficial effects in ischemic stroke. Here, we investigated the effects of genetic deficiency of COX-2 or inhibition of COX-2 with its highly selective inhibitor, CAY10404, on BBB damage and hemorrhagic transformation in a transient focal cerebral ischemia model in mice. Since IgG or Hb are barely detected in the non-injured brain due to the barrier function of an intact neurovascular unit, stroke-induced BBB damage will facilitate the leakage of these plasma proteins into the ischemic area. Our results showed that levels of IgG and Hb were significantly increased in the ischemic cerebral cortex of wild-type mice compared to sham-operated group, while the increases in these two plasma proteins were dramatically reduced in the COX-2^−/−^ mice or with post-ischemic treatment of CAY10404, suggesting that genetic deletion or pharmacological inhibition of COX-2 could attenuate stroke-induced BBB damage and hemorrhagic transformation. These findings are in line with previous reports that COX-2 deficient mice show smaller infarct volume than wild-type animals after transient MCAO (15, 18), whereas overexpression of COX-2 exacerbate the stroke outcomes (17).

Cellular components of the neurovascular unit, such as brain microvascular endothelial cells, pericytes, astrocytic end feet, neuronal processes, and perivascular microglia act in concert to provide a physiological barrier that guarantees brain homeostasis (41, 45, 46). Structural components of the basal lamina, tight junctions (TJs) and adherens junctions (AJs) play key roles in the maintenance of the integrity of the BBB (41). To explore the underlying structural components responsible for the COX-2-mediated BBB disruption in ischemic stroke, ZO-1 and occludin (two critical TJPs) as well as the basal lamina protein, collagen IV, were measured in ischemic brain of COX-2^+/+^ and COX-2^−/−^ mice since they are important in the maintenance of BBB integrity (40, 41). We found that levels of ZO-1, occludin and collagen IV were dramatically decreased in the ischemic cerebral cortex of wild-type mice as compared with sham-operated animals, which is consistent with previous reports showing that loss of specific TJPs or basal membrane proteins is associated with BBB opening (46–48). Importantly, genetic deletion of COX-2 prevented the stroke-induced loss of these critical structural proteins of the BBB and post-ischemic treatment with CAY10404 also significantly reduced the TJPs breakdown. In agreement with a previous report showing a decrease of JAM-A expression associated with BBB breakdown in a rat cortical cold injury model (44), we found a dramatic decrease of JAM-A protein levels, another BBB structural component, in ischemic cortex at 24 h of reperfusion in wild-type mice subjected to transient MCAO. Stroke-induced the loss of JAM-A was significantly restored in COX-2 deficient mice. Taken together, these findings strongly suggest that genetic deficiency or pharmacological inhibition of COX-2 mediates neurovascular protection in ischemic stroke through the preservation of specific BBB structural proteins.

MMP-9 is an important gelatinase that belongs to the MMP family involved in the degradation of extracellular matrix and tight junction proteins, thus leading to the BBB disruption (21, 23). Our data indicate that genetic deletion or pharmacological inhibition of COX-2 reduces MMP-9 production in stroke, which reveals for the first time that COX-2 activity may contribute to MMP-9 expression and activation in the ischemic brain, and the resulting BBB damage. However, it is not clear which downstream effectors of COX-2 mediate MMP-9 expression/activation in ischemic stroke. Previous studies from our group and others have demonstrated that COX-2 levels are increased in the ischemic brain, which is associated with the generation of large amounts of PGE_2_ (6, 10, 12), one of the major proinflammatory prostanoids formed by the COX-2 pathway. In support, neuronal overexpression of COX-2 in mice results in a dramatic increase (~10-fold) in the brain PGE_2_ levels compared with wild-type mice, which results in a marked increase in stroke volume (17). Conversely, genetic deletion or pharmacological inhibition of COX-2 results in smaller infarct volume, together with decreased levels of PGE_2_ in the ischemic brain after stroke (15, 18). Currently, there is no direct evidence showing that COX-2-derived PGE_2_ contributes to the MMP-9 activation and the resulting BBB damage in ischemic brain. However, the COX-2/PGE_2_ pathway has been widely reported to be involved in the upregulation of MMP-9 in cancer cells and primary monocytes/macrophages (25–28, 49–52). In the pancreatic cancer cell line, BxPC-3, inflammatory stimuli, such as lipopolysaccharides (LPS) or tumor necrosis factor alpha (TNF-α) significantly increase the expression of COX-2, PGE_2_, and MMP-9, and siRNA knockdown of COX-2 or treatment with the COX-2 selective inhibitor NS398 significantly reduces the increase in MMP-9 expression (28). In the human leukemic T cell line, HSB.2, PGE_2_ directly induces MMP-9 transcription, an effect that is mediated by the EP3 subtype of PGE_2_ receptor (49). The addition of PGE_2_ to macrophage cultures stimulates the expression of both urokinase-type plasminogen activator and MMP-9 (50). COX-2-dependent extracellular matrix-induced MMP-9 expression by macrophages is mediated by the PGE_2_ receptor subtype EP4 (51). Similarly, a previous report in monocytes demonstrated that MMP-9 production and activity are induced by PGE_2_ through an EP4-mediated mechanism (52). More recently, we found that genetic deletion or pharmacological inhibition of the PGE_2_ receptor EP1 reduces cerebral infarction, BBB damage and hemorrhagic transformation in an experimental ischemic stroke model, and these neuroprotective effects are associated with decreased MMP-9 levels and activity (29). Taken together, a large body of evidence suggests that MMP-9 is involved in COX-2/PGE_2_-mediated BBB damage in ischemic stroke.

The source of MMP-9 produced in ischemic brain is still controversial. Infiltrating neutrophils are believed to be the major source of active MMP-9 in cerebral ischemia (53–56). Neutrophils are present early in the ischemic tissue contributing to reperfusion injury (55–57). Our data indicate that myeloperoxidase (MPO, an indicator of peripheral neutrophil infiltration), neutrophil elastase (NE), and lipocalin-2 (LCN2, also known as neutrophil gelatinase-associated lipocalin) are markedly increased in the ischemic brain, and that the increased levels of these markers of neutrophil infiltration are significantly reduced in COX-2^−/−^ mice. This suggests that reduced neutrophil infiltration may be involved in limiting MMP-9 production, thus resulting in less BBB damage in COX-2^−/−^ mice following stroke. As an alternative interpretation, it is also possible that decreased MMP-9 levels/activity following COX-2 blockade is due to reduced activation of microglial cells and astrocytes, which would result in BBB protection after stroke (21, 23, 58–60). Less BBB damage in COX-2^−/−^ mice or in animals treated with the COX-2 inhibitor would limit the infiltration of MMP-9-laden neutrophils into the damaged brain (45, 61). Future studies are needed to explore the causal link between BBB damage, increased MMP-9 levels and neutrophil infiltration in the context of ischemic stroke, as well as determining how blockade of COX-2-dependent pathways modulates the activation of glial cells and neutrophils to protect the BBB after ischemic injury.

Neutrophil elastase and lipocalin-2 are two critical inflammatory mediators linked to neurovascular injury in stroke. NE belongs to the chymotrypsin-like family of serine proteases and plays an important role in immunity and host defense. This protease is mainly produced by neutrophils. Under pathological conditions, excess production of NE could result in tissue damage by degrading the extracellular matrix (62, 63). We found a significant increase in the levels of NE in the ipsilateral cerebral cortex at 24 h following transient focal cerebral ischemia, which is in line with the results from a previous study (64). Genetic deletion or pharmacological inhibition of NE significantly reduces infarct size, BBB disruption, vasogenic edema, as well as diminishes leukocyte adherence to the brain endothelium (64). LCN2 is another inflammatory mediator that is released from astrocytes, endothelial cells, and infiltrating neutrophils following ischemic brain injury (65–68). Consistent with these studies, we found that the levels of LCN2 increase dramatically in the stroked brain. Since both NE and LCN2 have been shown to mediate neuroinflammation and BBB damage in stroke (64–68), reduced levels of NE and LCN2 in COX-2 deficient animals could be a potential mechanism underlying the vasculoprotective effects of COX-2 blockade in stroke.

In summary, our data demonstrate the beneficial effects of genetic deletion or pharmacological inhibition of COX-2 on preservation of BBB integrity, maintenance of structural components of the neurovascular unit, reduction of MMP-9 expression/activity as well as limitation of neutrophil infiltration in a mouse model of transient focal cerebral ischemia. These results indicate that MMP-9 is one of the downstream effectors in COX-2/PGE_2_-mediated BBB damage in ischemic stroke. Targeting the COX-2/MMP-9 pathway could represent a promising strategy to reduce brain injury and preserve the integrity of the BBB following stroke.

## Data Availability Statement

The raw data supporting the conclusions of this article will be made available by the authors, without undue reservation.

## Ethics Statement

The animal study was reviewed and approved by University of Florida Institutional Animal Care and Use Committee (protocol # 201607934).

## Author Contributions

CY, YY, KD, and EC-J performed the experimental procedures. CY, GR, and EC-J designed the research and planned all the experiments. CY, KD, and EC-J analyzed the data and prepared the figures. CY and EC-J wrote the article. EC-J conceived and led the project. All the authors read and approved the final version of the manuscript.

## Conflict of Interest

The authors declare that the research was conducted in the absence of any commercial or financial relationships that could be construed as a potential conflict of interest.

## Abbreviations

COX-2: cyclooxygenase-2
NSAIDs: non-steroidal anti-inflammatory drugs
AA: arachidonic acid
PGE_2_: prostaglandin E_2_
COX-2^−/−^: COX-2 knockout
BBB: blood-brain barrier
MMP-9: matrix metalloproteinase-9
TJPs: tight junction proteins
MCAO: middle cerebral artery occlusion
CCA: common carotid artery
ECA: external carotid artery
ICA: internal carotid artery
DMSO: dimethyl sulfoxide
TTC: 2,3,5-triphenyltetrazolium chloride
PBS: phosphate-buffered saline
RIPA: radioimmunoprecipitation
IgG: immunoglobulin G
Hb: hemoglobin
ZO-1: zonula occludens-1
JAM-A: junctional adhesion molecule-A
MPO: myeloperoxidase
NE: neutrophil elastase
LCN2: lipocalin-2
RFUs: relative fluorescence units
TJs: tight junctions
AJs: adherens junctions
MMPs: matrix metalloproteinases
LPS: lipopolysaccharide
TNF-α: tumor necrosis factor alpha.
